# *Astragalus membranaceus* Additive Improves Serum Biochemical Parameters and Reproductive Performance in Postpartum Dairy Cows

**DOI:** 10.3389/fvets.2022.952137

**Published:** 2022-07-11

**Authors:** Yinghao Huang, Qi Yan, Maocheng Jiang, Sheng Guo, Huiwei Li, Miao Lin, Kang Zhan, Guoqi Zhao, Jinao Duan

**Affiliations:** ^1^Institute of Animal Culture Collection and Application, College of Animal Science and Technology, Yangzhou University, Yangzhou, China; ^2^Jiangsu Collaborative Innovation Center of Chinese Medicinal Resources Industrialization, National and Local Collaborative Engineering Center of Chinese Medicinal Resources Industrialization and Formulae Innovative Medicine, Jiangsu Key Laboratory for High Technology Research of TCM Formulae, Nanjing University of Chinese Medicine, Nanjing, China; ^3^Institutes of Agricultural Science and Technology Development, Yangzhou University, Yangzhou, China; ^4^Joint International Research Laboratory of Agriculture and Agri-Product Safety, the Ministry of Education of China, Yangzhou University, Yangzhou, China

**Keywords:** postpartum dairy cows, *Astragalus membranaceus*, plant additives, postpartum recovery, alternative to antibiotics

## Abstract

The purpose of the study was to assess the recovery, immune function, and breeding efficiency of postpartum dairy cows fed *Astragalus membranaceus* (AM) as a feed additive. The experiment used a completely randomized design. Cows were randomly assigned to two groups: (1) Control group fed total mixed ration (TMR; CON group, *n* = 15); (2) AM group fed TMR and AM (AM group, *n* = 15). The AM group was fed 675 g/day. The experimental results showed that compared with the CON group. The breeding interval of the AM group of dairy cows had a tendency to shorten (0.05 < *p* < 0.1). Plasma viscosity (PV), Plasma fibrinogen (FIB), the red cell aggregation index (TRCAI), Calcitonin (CT), Immunoglobulin M (IgM), and Luteinizing hormone (LH) results of AM group showed a time-treatment interaction (*p* < 0.05). Furthermore, the result of the study revealed that feeding AM as feed additives to dairy cows during the postpartum period had positive effects on wound recovery, immune function, endocrine regulation, and breeding efficiency.

## Introduction

The perinatal period is a special and important part of the production cycle of dairy cows because the health of dairy cows during the perinatal period will directly affect the subsequent milk production and breeding (1). The physical health of cows during the perinatal period is highly challenged due to enormous pressure from calving and subsequent lactation (2). Cows within the perinatal period experience perinatal weakness or diseases such as metritis, postpartum paralysis, and various inflammations arising from wound infection (3). These diseases possess serious harm to the production of dairy cows, and dairy cows may even be culled (4). The administration of antibiotics has long been the only option employed to help cows through the perinatal period, especially the difficult post-perinatal period (5). However, the large-scale use of antibiotics, notably in the animal feed and food industry (6), is of major concern due to the presence of residues of antibiotics within animal products (meat, milk, and dairy products) which pose a threat to human health (7). Recently within the food industry, consumers have been very critical and mostly oppose the purchase of foods containing antibiotics (8).

Plants usually contain a variety of bioactive substances with anti-inflammatory and bacteriostatic effects, such as flavonoids, phenols, and terpenes (9). Numerous studies have demonstrated the positive effects of plants and plant extracts on animal growth and health (10). *Astragalus membranaceus* (AM) is a leguminous plant widely distributed in temperate regions of the world. AM is also a widely used medicinal plant (11). The previous studies have shown that AM contains more than 100 active substances. The major phytoconstituents of Astragalus species with beneficial properties are saponins, flavonoids, and polysaccharides (12). AM is a plant with various effects such as antioxidant, anti-inflammatory, antiviral, and promoting wound recovery (13). There are around 170 saponins of the cycloartane- and the oleanane type (14). Astragaloside IV has anti-inflammatory, antioxidant, anti-aging, and preventing arteriosclerosis properties (15), and has the function of protecting the nervous system (14). Regarding its anti-infective properties, its mechanism may involve inhibition of viral replication (16). There are over 60 kinds of flavonoids compounds, such as isoflavones, rosetanes, flavonols, isoflavonols, and dihydroisoflavones (17); isoflavones are the main active substances (18). These flavonoids confers anti-inflammatory, antiviral (18), anti-infection, and anti-osteoporosis effects on AM (19). More than 30 kinds of *A. membranaceus* polysaccharides (APS) have been found in AM, and their pharmacological activities have been verified in both *in vivo* and *in vitro* studies. The main dextran and heteropolysaccharides have anti-inflammatory and immune-enhancing properties (20). The previous studies have shown that APS can directly, or insights inhibit the replication of a variety of animal viruses (21). AM fed to lambs had positive antioxidant effect and feed intake. Feeding AM extract specifically *A. membranaceus* polysaccharide to mice significantly improved their immunity (22). Furthermore, feeding fermented AM as an antibiotics replacement to laying hens promoted hens immunity, anti-inflammatory, and antioxidant effects (23). Feeding AM root powder to fattening sheep improved their antioxidant and immune function (24). AM has anti-inflammatory, antiviral, and immune-boosting effects. However, in the existing literature, there is scarcity of research assessing the potential use of AM to alleviate the negative impact associated with the difficult perinatal period experienced by dairy cows. It is, therefore, hypothesized that, the various bioactive substances contained in AM would help the uterus and body of dairy cows recover as soon as possible without the use of antibiotics. This would ensure that dairy cows can smoothly enter the next production cycle.

Thus, this experiment utilized an AM-based additive, which was fed to dairy cows in the post-perinatal period for 21 days.

## Materials and Methods

### Ethical Considerations

All Holstein bovines used in this research were strictly cared for in accordance with the principles of the Institutional Animal Care and Use Committee (IACUC) of Yangzhou University (SYXK (Su) 2016-0019).

### Source of *Astragalus membranaceus*

The raw material of traditional Chinese medicine used in this experiment was provided by the Nanjing University of Chinese Medicine. The main raw material was AM powder, which was processed into granular feed by Jiangsu Youxin Feed Company.

### Experimental Design, Animals, Diets, and Management

The experiment was conducted from October to December 2021 at the Experimental Base of Animal Nutrition and Feed Engineering Research Center, Yangzhou University (China). The experiment used a completely randomized design. Cows were randomly assigned to two groups: (1) Control group fed total mixed ration (TMR) (CON group, *n* = 15); (2) AM group fed TMR and AM (AM group, *n* = 15). All 30 experimental Holstein cows were first-born cows, and had similar body weight (613.4 ± 32.6 kg) and health status. The AM group supplement 675 g/day feed additives containing AM supplements are provided by the Nanjing University of Chinese Medicine and the composition of the AM is shown in Table 1. The composition of the TMR is shown in Table 2. The trial commenced on the day parturient cows calved and followed by the feeding of AM feed additive. The duration of the experiment was 21 days. All 30 Holstein cows had free access to TMR (105%) and freshwater. Holstein cows were fed at 8:00 and 14:00 and at 21:00. The time of milking was the same as the time of feeding.

**Table 1:** *Astragalus membranaceus* (AM) feed additive contents.

**Items**	**Content, %**
Astragalus membranaceus powder	20
Brown sugar	8
Yeast powder	10
Corn	34
Soya bean meal	10
Salt	1
Palm oil	2
Flax meal	10
Bran	5
Total	100

**Table 2:** Basal diet formulations and nutritional contents (DM basis, %).

**Items**	**Content**
Alfalfa hay	8.39
Oat hay	6.43
Whole corn silage	52.18
Corn	8.1
Soya bean meal	10.8
Cotton seed meal	6.8
DDG	4.8
Premix^1^	2.5
Total	100
**Nutrient levels**	
CP	15.15
EE	3.75
NDF	33.51
ADF	21.72
Ash	7.12
Calcium	0.42
Phosphorus	0.25
NE_L_ (MJ/kg)^2^	6.14

*DDGS, distiller dried grains with soluble; CP, crude protein; EE, ether extract; NDF, neutral detergent fiber; ADF, acid detergent fiber*.

1 *Per kilogram of premix provide 55 mg of Mn as MnSO_4_; 62.5 mg of Zn as ZnSO_4_; 0.55 mg of Se as Na_2_SeO_3_; 75 mg of Fe as Fe_2_(SO_4_)_3_; 32 mg of Cu as CuSO_2;_ 1.00 mg of I as KI; 0.55 mg of Se as Na_2_SeO_3_; 0.40 mg of Co as CoCl_2_; 1,100,000 IU of Vitamin A; 270,000 IU of Vitamin D; 200,000 mg of Vitamin E; 4,200 mg of Vitamin K3*.

2 *Predicted values from NRC (2001) model (25)*.

### Data Collection and Analysis

The experiment began after the parturition of the experimental cows. The body measurements were carried out on the 7th, 14th, and 21st days postpartum. The body weight of the cows was calculated based on body measurement. The lochia discharge of the cows was observed and recorded every day. The breeding situation of experimental cows was monitored by recording the success rate of breeding cows within 60 days after giving birth.

Two sets of vacuum collection tubes (non EDTA and EDTA) was used to collect 10 ml of tail vein blood on the 0th, 1st, 7th, 14th, and 21st days postpartum. Non EDTA set of tubes were centrifuged immediately at 3,000 rpm at 4 °C for 30 min. The supernatant was collected and stored at −80 °C for subsequent analysis.

The EDTA tube was promptly dispatched to Yangzhou University Animal Hospital for blood routine indicators.

After all experimental bovine serum has been collected, the cow serum samples were be tested for platelet count (PLT), plasma viscosity (PV), plasma fibrinogen (FIB), the red cell aggregation index (TRCAI), IL-2, IL-6, C-reactive protein (CRP), Calcitonin (CT), IgM, IgG, IgA, prostaglandin 2α (PGF2α), Gonadotropin-releasing hormone (GnRH), prolactin (PRL), estradiol (E2), luteinizing hormone (LH), follicle stimulating hormone (FSH), and progesterone (P).

Clotting time serum sample testing was done by Beijing Huaying Biotechnology Company.

### Statistical Analysis

The analysis was performed using SPSS statistical software, version 20.0 (IBM Corporation, Armonk, New York, USA) and the model included the random effect of cow, period, and treatment sequence and the fixed effects of a covariate, treatment, day of treatment, and their interaction. In addition, **Table 4** used an independent sample *t*-test. Different letters indicate significant differences (*p* < 0.05), and the same letters indicate no significant differences (*p* > 0.05).

## Results

### Cow Performances and Reproductive Performance

No interaction between time and treatment was observed in the results of this trial in the measurement of chest circumference (*p* > 0.05, Table 3). Nonetheless, after treatment, the AM group showed significantly higher results than the CON group in terms of bust circumference (*p* < 0.05, Table 3). A similar trend was observed with respect to the calculated body weight (*p* < 0.05, Table 3). However, no interaction of time and treatment was observed in the results of body oblique length. This finding is similar to that of other scholars, thus adding AM to the diet can increase the energy intake of animals. This relieved the cows negative energy balance after birth, but did not stop it. In terms of reproductive performance (Table 4), the time from calving to pregnancy in the AM group was smaller than in the CON group, and there was a downward trend (*p* = 0.07). This result is vital and directly reflects the effect of this experiment in helping to shorten the mating interval.

**Table 3:** Effects of AM on body condition of dairy cows in perinatal period.

**Items**	**Treatments**						**SEM**	***p*** **-value**		
	**CON**			**AM**				**Time**	**Treat**	**Interaction**
	**7 days**	**14 days**	**21 days**	**7 days**	**14 days**	**21 days**				
Chest circumference (m)	2.10	2.08	2.05	2.12	2.10	2.09	0.03	0.07	<0.01	0.79
Oblique length (m)	1.53	1.52	1.52	1.57	1.56	1.55	0.02	0.89	0.23	0.99
Weight^1^ (kg)	611.22	594.11	575.45	630.59	619.34	605.62	13.4	0.22	<0.01	0.93

1 *Wight was calculated, and the rest were measured*.

*Weight = chest circumferenc × chest circumferenc × oblique length × 90*.

**Table 4:** Time from calving to next pregnancy in AM and CON.

**Items**	**Treatments**		**SEM**	***p*** **-value**
	**CON**	**AM**		
Calving to pregnancy (day)	126.10	112.00	7.21	0.07

### Coagulation Index

**Figure 1:**
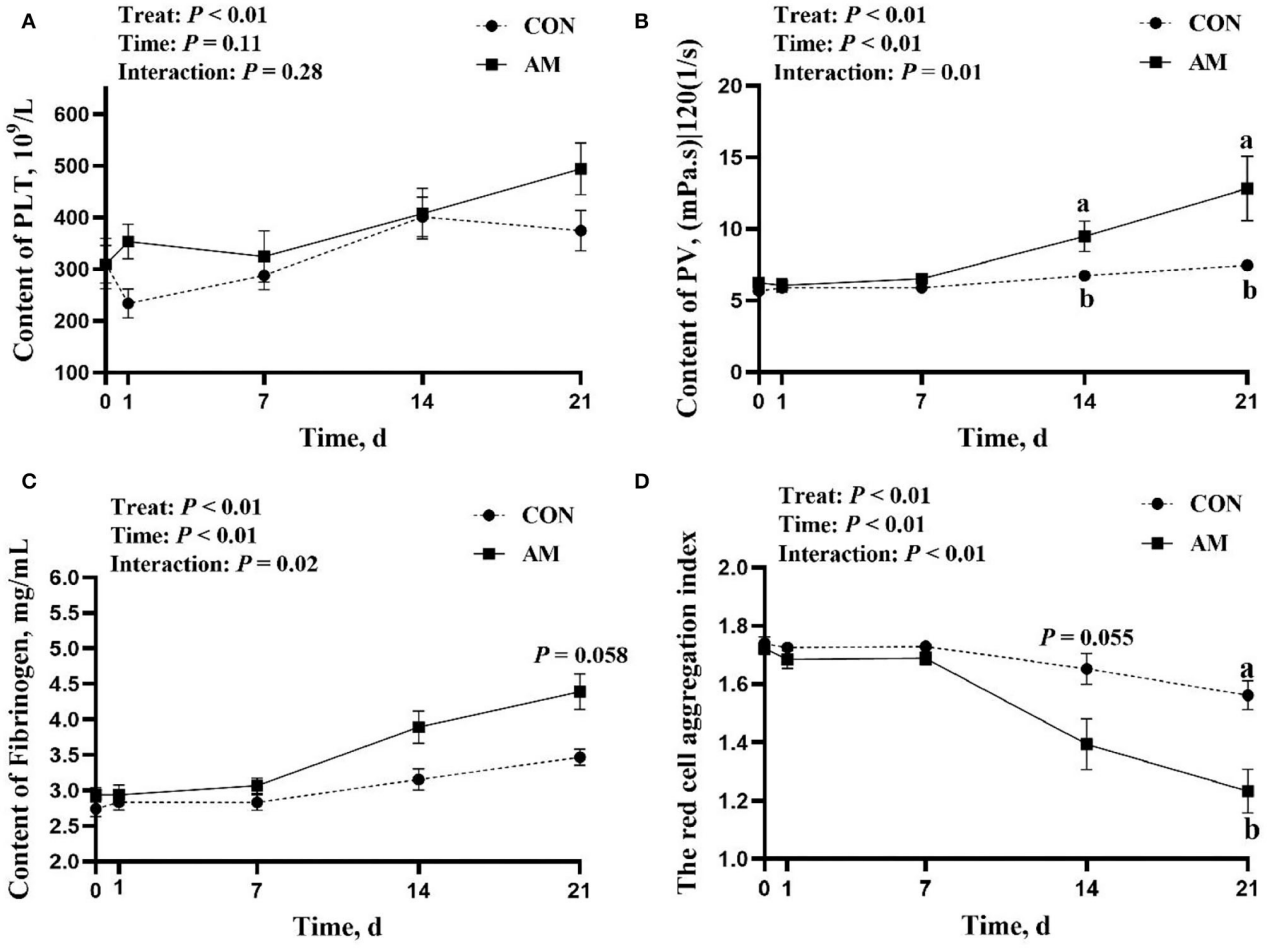
Coagulation index of CON and *Astragalus membranaceus* (AM) in Holstein dairy cows at 21 days postpartum. **(A)** Platelet count, **(B)** Plasma viscosity, **(C)** Plasma fibrinogen, and **(D)** The red cell aggregation index. Data shown are means ± SEM. Different lowercase letters indicate significant differences.

The results of this experiment show that AM plays a certain role in helping blood coagulation and wound recovery in perinatal dairy cows. The results show that the interaction of PV, FIB, and TRCAI in time and treat is significant (*p* < 0.05). The PV and TRCIA of AM group were significantly higher than the CON group at 14 and 21 days of AM (*p* < 0.05, Figures 1B,D). TRBIC trend was higher in AM group than in CON group for 14 days (*p* = 0.055). However, this difference did not appear in the early stages of the experiment, that is, from the start of the experiment to the 7th day of the experiment. The reason for this phenomenon may be that drugs commonly require continuous use to have a certain effect, so this is a normal phenomenon. This phenomenon also appeared in the FIB result. There was no significant difference between the AM group and the CON group in the early stage of the experiment. However the AM group was significantly higher than the CON group when FIB was fed AM for 21 days (*p* < 0.05, Figure 1C). The results of this study showed that there was no interaction between PLT time and treatment in the AM group (*p* > 0.05, Figure 1A).

### Inflammatory Factor and Immunoglobulin

**Figure 2:**
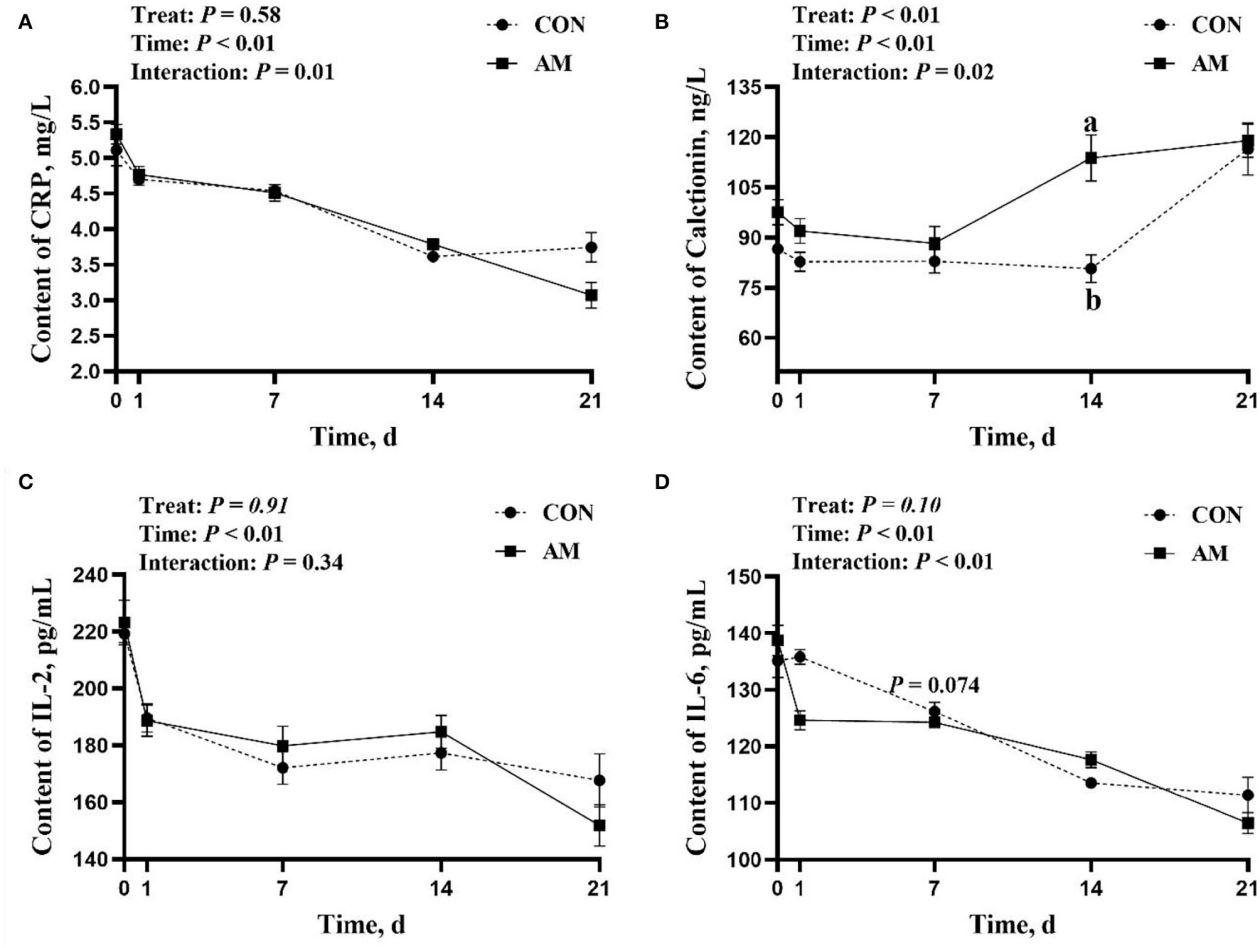
Inflammatory factor of CON and AM in Holstein dairy cows at 21 days postpartum. **(A)** C-reactive protein, **(B)** Calcitonin, **(C)** IL-2, **(D)** IL-6. Data shown are means ± SEM. Different lowercase letters indicate significant differences.

The results showed that CRP, CT, and IL-6 had time-treat interaction in the AM (*p* < 0.05, Figures 2A–D). Among them, the interaction effect of CRP was due to the effect of time, and the treatment did not bring about a significant difference. The AM group had a higher trend than the CON group when CT was fed for 14 days (Figure 2B). However, this difference was only observed in the monitoring results on day 14. IL-6 in the AM group tended to be higher than the CON group when fed for 7 days (*p* = 0.074, Figure 2D). This trend is also appeared only on the 7th day.

**Figure 3:**
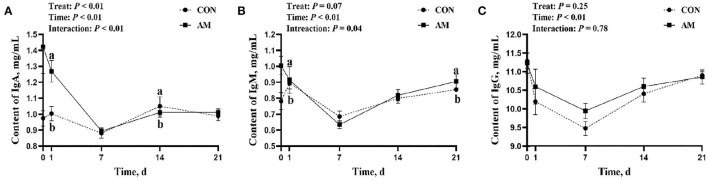
Immunoglobulin of CON and AM in Holstein dairy cows at 21 days postpartum. **(A)** Immunoglobulin A (IgA), **(B)** Immunoglobulin M (IgM), **(C)** Immunoglobulin (IgG). Data shown are means ± SEM. Different lowercase letters indicate significant differences.

The effect of this test on immunoglobulin is shown in the following aspects. There was a time–treat interaction for IgA (*p* < 0.05, Figure 3A), with CON being significantly higher than AM at 1-day feeding (*p* < 0.05). However, this significance faded in subsequent monitoring, only to reappear at 14 days. The AM group was significantly higher than the CON group at 14 days of feeding (*p* < 0.05). There was an interaction trend between time and treat of IgM in AM group (*p* = 0.04, Figure 3B). The AM group was significantly higher than the CON group at both 1 and 21 days of feeding (*p* < 0.05). No differences were seen at other monitoring times points.

### Reproductive Hormones

**Figure 4:**
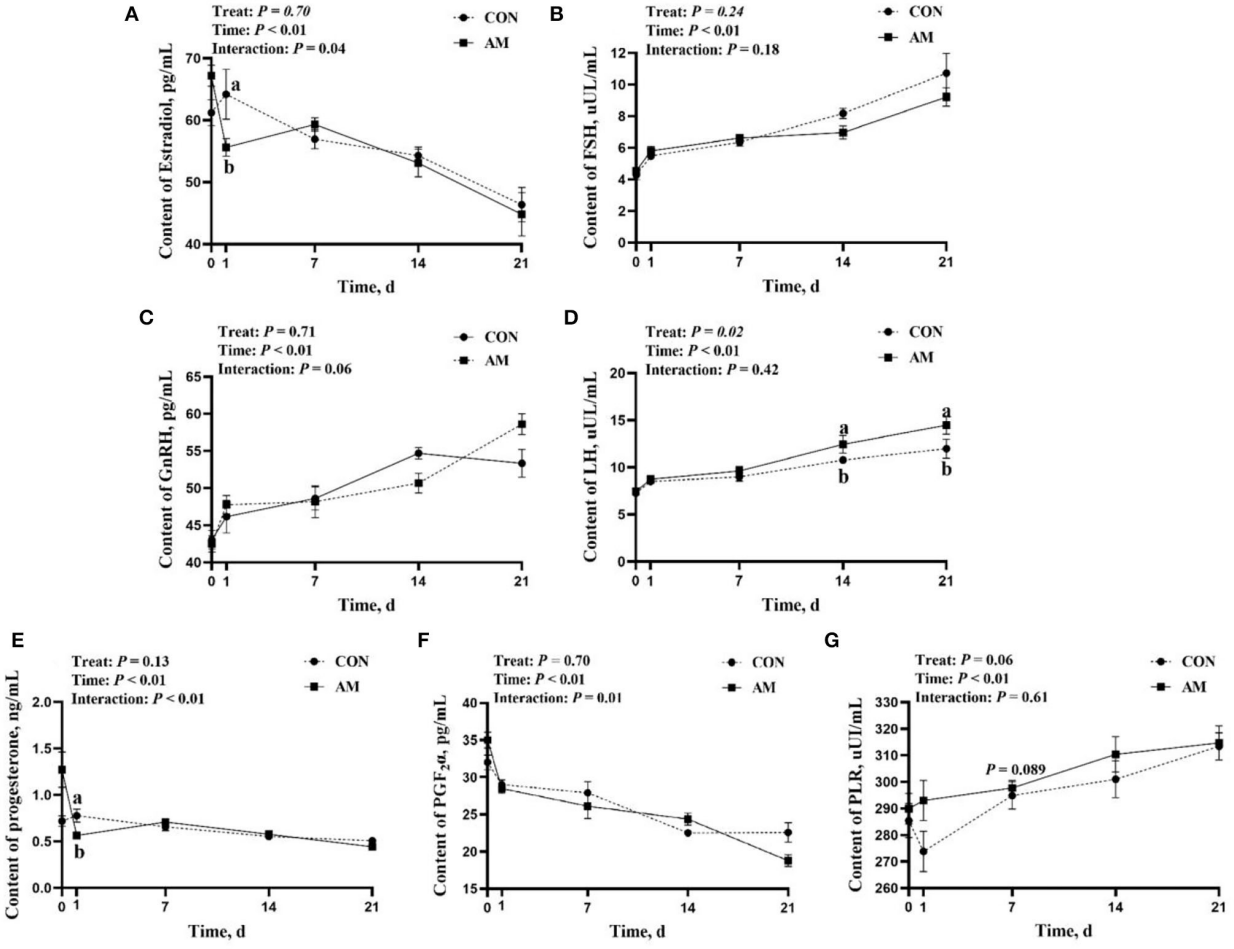
Reproductive hormones of CON and AM in Holstein dairy cows at 21 days postpartum **(A)** Estradiol, **(B)** Follicle stimulating hormone, **(C)** Gonadotropin-releasing hormone, **(D)** Luteinizing hormone, **(E)** Progesterone, **(F)** Prostaglandin 2α, **(G)** Prolactin.

The results showed that E2, P, and PGF2α had time–treat interaction after AM feeding (*p* < 0.05, Figures 4A–F). A similar trend was observed with GnRH (*p* = 0.06, Figure 4C). At the beginning of the experiment, there was no difference in the LH of AM and CON groups. Nevertheless, LH of AM group was significantly higher than the CON group on days 14 and 21 of monitoring (*p* < 0.05, Figure 4D). There was an upward trend in AM group when PLR was fed for 7 days (*p* < 0.05, Figure 4G). No differences in PLR were found in monitoring at other time points.

## Discussion

The AM is generally used by mixing a variety of plants in a certain proportion. However, in the AM used in this experiment, its main component is AM powder. Some experiments have shown that adding AM to the basal diet will increase animal feed intake (24), improve animal body condition and increase body weight (26). Similarly, in this study, cows fed AM exhibited a certain advantage in body condition. Although postpartum body fat mobilization, body weight, and body indexes declined in all experimental cows groups, cows fed AM experienced a smaller decrease compared to the CON group. This is primarily due to the fact that the cows are under intense stress after giving birth, and the feed intake is at a low level, leading to a negative energy balance and an overall poor body condition of the cows (27). That notwithstanding, the results still indicated that adding AM alleviated the weight loss of dairy cows to a certain extent during this period. While this does not completely stop the cows from losing weight, it relatively helps to reduce the stress cows experience during postpartum period. A cow in the better physical condition is always more resistant to risks than a cow in poor physical condition.

Calving is a painful process, accompanied by heavy bleeding. This severe bleeding is manifested not only in external wounds but also in internal bleeding caused by the rupture of blood vessels in the body (28). Hence, accelerating blood clotting can help cows recover from vascular damage in the body, thereby relieving a series of postpartum diseases caused by massive bleeding in cows. In the present study, a number of indicators revealed that adding AM to dairy cow feed in the postpartum period could accelerate blood coagulation in dairy cows and help cows recover wounds. At the same time, the TRCIA value of the AM group was lower than that of the CON group (29), which infer that this blood coagulation is not due to blood coagulation originating from the aggregation of red blood cells (30). In other words, the AM used in this study did not induce thrombus while promoting blood coagulation in cows. The result of a previous study involving rat-fed AM-containing feed is consistent with the conclusions of this study (31). This is also in line with the theory of qi and blood in traditional Chinese medicine (32). However, the mechanism by which AM promotes blood coagulation is yet to be elucidated.

The AM is a plant rich in various active substances, such as flavonoids, polysaccharides, and terpenoids (13). These active substances have various anti-inflammatory properties and antioxidant functions (33). After calving, the immunity of the dairy cows is at its lowest point, and concurrently, there are huge wounds within the reproductive tract (34). These wounds can be easily infected, causing reproductive tract inflammation (35). The results of this experiment showed that both IL-6 and CRP in the AM group were significantly lower than those in the CON group. IL-6 and CRP are important inflammatory markers. This difference in the levels of inflammatory markers means that adding AM to postpartum feeds can effectively help animals fight postpartum inflammation (36). The cows are able to recover from this weakening period after parturition as soon as possible. For the anti-inflammatory mechanism of AM, studies have shown that extracts of AM inhibit gene expression of iNOS and TNF-α at the transcriptional level (37), interfere with the NF-κB signaling pathway, and then inhibit the expression of IL-6 and CRP (38).

The level of CT, to a certain extent, can indicate the degree of bacterial infection experienced by the animal (36). In certain period of dairy cows late perinatal stage, the content of CT has other vital connotations. Cows enter the lactation stage immediately after calving, and a large amount of calcium enters the mammary gland for milk production (39). During this stage, cows tend to mobilize bone calcium, resulting in a decreased calcium content of bodily tissues (40). Excessive utilization of bone calcium will cause osteoporosis in dairy cows, which can cause skeletal diseases and bring huge losses to production (41). CT has the function of inhibiting the activity of osteoclasts and promoting the activity of osteoblasts (42). CT plays a huge role in calcium homeostasis in dairy cows, and the level of CT content determines to a certain extent whether dairy cows can rapidly recover from postpartum weakness. Studies have demonstrated that the CT content of normal cows after calving is 3.9 times that of paralyzed cows (43). The results of this study showed that the CT content of dairy cows in the AM group was significantly higher than that in the CON group. This means that the inclusion of AM in dairy cows diets had a positive effect on postpartum dairy cow's bone health. The previous studies have shown that various extracts in AM can improve the immune activity of animal bodies (44). The previous study had shown that the inclusion of AM in mice diet is capable of treating skeletal disease due to the ability of active substances in AM to act on the RANKL-RANK pathway (45). This is in agreement with the effect of AM used in this study on skeletal diseases of dairy cows.

Immunoglobulin is a self-produced anti-toxic factor. Many studies have proved that it plays an important role in animal immunity (46). APS is the most important active substance in AM. APS regulates the body's immune function and the release of immunoglobulins by increasing the immune organ index (47), stimulating the release of immune factors and affecting immune signaling (48). In this experiment, after adding AM for a period of time, the IgM and IgA in the serum of the experimental animals were significantly higher than those in the CON group. This implies that the AM used in this experiment can promote the production of immunoglobulins in dairy cows and strengthen the immunity of animals. Under natural conditions, the immunoglobulin level of dairy cows during the perinatal period will be at a low level (4). The addition of AM in this experiment can effectively alleviate this situation, which is of special significance. Similar results were reported with pigs fed a similar traditional Chinese medicine additive. The immunoglobulin in the treatment group was significantly higher than that in the control group, which confirms the effect of traditional Chinese medicine feed additives in improving the immunity of animals (49).

Compared with the CON group, the breeding rate of AM group had an upward trend, which is a manifestation of the effect of traditional Chinese medicine in this experiment. Animals' hormone levels tend to change significantly in the weeks following delivery (50). LH level of dairy cows tended to increase, E2 level remained for a period of time, and the P level decreased in short time (51). The previous studies have shown that GnRH stimulates the release of LH after parturition in dairy cows (52). In this study, the LH of AM group was significantly higher than that in CON group. LH is an important hormone that regulates the estrus cycle of dairy cows and promotes ovarian recovery. The increase of LH will stimulate the release of other sex hormones to promote positive feedback, and under the combined action of multiple sex hormones, help cows restore ovarian function, and enter the next follicle maturation period (53). Compared with the CON group, there was an upward trend in PRL in the AM group. PRL is an important hormone that promotes lactation and mammary gland development in animals. Research has showed that PRL could promote the proliferation of cow mammary epithelial cells (54).

## Conclusions

In conclusion, the results of this study have shown that the inclusion of AM positively impacts the reproductive performance, immunity, and endocrine of dairy cows during the perinatal period. This provides new ideas that can be used by dairy farmers to ensure the safe transition of cows during the perinatal period and to reduce antibiotic dependence.

## Data Availability Statement

The original contributions presented in the study are included in the article/supplementary material, further inquiries can be directed to the corresponding authors.

## Ethics Statement

All Holstein bovines used in this research were strictly cared for in accordance with the principles of the Institutional Animal Care and Use Committee (IACUC) of Yangzhou University [SYXK (Su) 2016-0019]. Written informed consent was obtained from the owners for the participation of their animals in this study.

## Author Contributions

GZ and JD designed the whole experiment and verified the validity of experiment and checked the results. YH, SG, QY, and HL performed the experiment, including chemical analysis and statistical analysis. YH, MJ, and KZ worked on the manuscript. YH, ML, and KZ participated in the experiment design and gave valuable advice. All authors have read and approved the final version of this manuscript.

## Funding

This study was supported by the National Natural Science Foundation of China (No. 31972589), the earmarked fund for CARS36, the Central Government's Major Increase and Decrease Project (2060302), and Key Projects of Ningxia Key Research and Development Program (2020BFH02013).

## Conflict of Interest

The authors declare that the research was conducted in the absence of any commercial or financial relationships that could be construed as a potential conflict of interest.

## Publisher's Note

All claims expressed in this article are solely those of the authors and do not necessarily represent those of their affiliated organizations, or those of the publisher, the editors and the reviewers. Any product that may be evaluated in this article, or claim that may be made by its manufacturer, is not guaranteed or endorsed by the publisher.
